# Effects of basic type of intermittent exotropia on myopic shift in children: a 12-month observational study

**DOI:** 10.3389/fped.2024.1513062

**Published:** 2025-01-06

**Authors:** Jing-Xin Li, Xiang-Xiang Liu, Jie Hao, Hui-Xin Li, Qiong-Yue Zhang, Yi-Yang Zhao, Yu-Meng Wang, Lei Li, Jing Fu

**Affiliations:** Beijing Tongren Eye Center, Beijing Tongren Hospital, Capital Medical University, Beijing Key Laboratory of Ophthalmology & Visual Sciences, Beijing, China

**Keywords:** intermittent exotropia, myopic shift, intermittent exotropia surgery, axial elongation, accommodation and convergence

## Abstract

**Background:**

To investigate the effect of basic intermittent exotropia (IXT) on myopic shift in children during 12-month follow-up.

**Methods:**

165 children aged 4–15 years were recruited prospectively in this study and divided into 3 groups: Group A, consisted of 64 patients with basic IXT without surgery; Group B, consisted of 51 patients 1-month after IXT-corrected surgery; and Group C, consisted of 50 patients without any form of strabismus. All patients underwent assessments of spherical equivalent (SE), axial length (AL), exodeviation, and binocular function relating to accommodation and convergence. Examinations were conducted at baseline and 12-month. SE and AL changes were compared among groups. Univariate and multivariate linear analyses were employed to investigate the association between myopic shift and IXT, as well as other clinical parameters.

**Results:**

Three groups showed comparable ages, genders and SEs at baseline (all *P* > .05). During 12-month follow-up, the rate of myopic shift varied among groups. Significant differences in SE progression (*P* = .006) and AL elongation (*P* = .014) between Group A and Group C were observed. Although SE progression and AL elongation in Group B were less than Group A, no significant differences were found (*P* = .125; *P* = .038). In the multivariate analysis, increases in exodeviation angle were significantly associated with both SE progression (*β* = 0.010, *P* = .041) and AL elongation (*β* = −0.005, *P* = .026). Each one prism diopter increase in the exodeviation angle was correlated with a 0.01D SE progression and a 0.005 mm AL elongation.

**Conclusions:**

Children with basic IXT exhibited faster myopia shift compared to those without strabismus. Although surgical correction of strabismus appeared to slow this process, the effect was not statistically significant. Furthermore, greater increase in exodeviation angle was associated with higher rate of SE progression and AL elongation.

**Trial registration:**

The study was approved by the Ethics Committee of Beijing TongRen Hospital (approved number: TRECKY2020-142, approved date: 2020.10.30).

## 1 Introduction

Refractive errors, particularly myopia, are the main causes for approximately 20% of blindness worldwide (1). It is estimated that myopia will affect 50% of the world's population by 2050. In addition, myopia affects a significant number of school-aged children, particularly in East Asian populations. Resnikoff et al. (2) and Zheng et al. (3) reported that up to 80% of students developed myopia by the time they graduated from high school. Recently, there were several large ongoing trials targeting the premyopic phase [−0.50 diopters (D) to +0.75D] (4) of children (5, 6). It was reported that children with premyopia were at particular risk of complications associated with myopia (5, 7), including myopic maculopathy (8), cataracts (9), and open-angle glaucoma (10). In addition, it is worth noting that the intermittent exotropia (IXT), another common eye disease affecting 0.12%–3.90% of children in Asia (11–14), can be co-existing with myopia (15). However, myopic progression in patients with IXT has not been rigorously studied. Ekdawi et al. (15) reported a higher incidence (more than 90%) of myopia in IXT patients. While other studies provided various conclusions about the differences in rate of myopic progression among children with IXT, without IXT and after IXT surgeries (16–18). Therefore, the correlation between myopia and IXT is still controversial.

Recent studies have reported that IXT may contribute to the myopic progression in children (15, 19, 20). The differences in accommodation and convergence between IXT patients and healthy subjects may provide potential explanations. The International Myopia Institute (IMI) has extensively reviewed the role of accommodation in myopic progression, highlighting the correlations between sustained near work demanding high levels of ocular accommodation and the myopic development (21, 22). Children with IXT may experience increased convergence accommodation as they exert more effort to control exodeviation (23, 24). Additionally, brief periods of sustained accommodation could lead to transient axial elongation (25–27). Given that excessive accommodation has been implicated as a risk factor of myopia (21, 22) and excess convergence accommodation was stimulated in IXT children (23, 24), it becomes crucial to explore “whether the intermittent exodeviation influences the progression of refractive errors in children and whether the accommodation and convergence in binocular vision function are critical factors in this process” were worth investigating.

This prospective study investigates the influence of basic type of IXT (28) on the myopic shift in children. To our knowledge, this is the first study to reveal the possible association between strabismus development and myopic shift through accommodation- and convergence-related binocular functional parameters. It aims to identify potential clinical indicators of myopic shift in children with IXT, and underscores the importance of incorporating the management of binocular vision anomalies into comprehensive myopia control strategies.

## 2 Materials and methods

### 2.1 Study population and design

This prospective study enrolled out patients visiting the Department of Strabismus & Pediatric Ophthalmology of Beijing TongRen Hospital for the first time between September 2022 and December 2022. The inclusion and exclusion criteria are detailed in Table 1.

**Table 1:** Inclusion and exclusion criteria.

Inclusion criteria
1. 4–15 years old; 2. For Group A and Group B before IXT-corrected surgery, children were diagnosed with basic type of IXT according to Burian^a^ (28) and exodeviation angle at near and distance was both more than 15PD; For Group B, children achieved straight ocular alignment at 1-month visit after surgery (exophoria less than 5 prism diopters); For Group C, children did not have a diagnosis of any form of strabismus; 3. No previous ophthalmic surgery for any reason, including strabismus surgery or botulinum injection; 4. No previous treatment for myopia other than monofocal refractive correction; 5. Subjects could cooperate with ophthalmologic examinations during study period, the guardians understood content of this research and were willing to sign the informed consent forms.
Exclusion criteria
1. Other kinds of strabismus such as vertical deviation of more than 5PD, dissociated vertical deviation (DVD), A- or V- pattern strabismus, paralytic or restrictive exotropia; 2. Ocular or neurologic disorders (e.g., attention deficit hyperactivity disorder); 3. Amblyopia (monocular distant vision worse than 20/25), anisometropia greater than 2.0D or astigmatism greater than 2.0D; 4. Refractive errors exceeding −6.00D (myopia) or +0.75D (hyperopia); 5. Accepted myopia progression management (accommodative training, low-dose atropine, orthokeratology lenses, defocusing spectacles); 6. Accepted treatment for exotropia (convergence training, overminus lens therapy); 7. High myopic family history.

IXT, intermittent exotropia; PD, prism diopters; D, diopters.

^a^ Patients were classified as having basic type of intermittent exotropia if the difference between exodeviation at distance and near was within 10PD.

**Figure 1:**
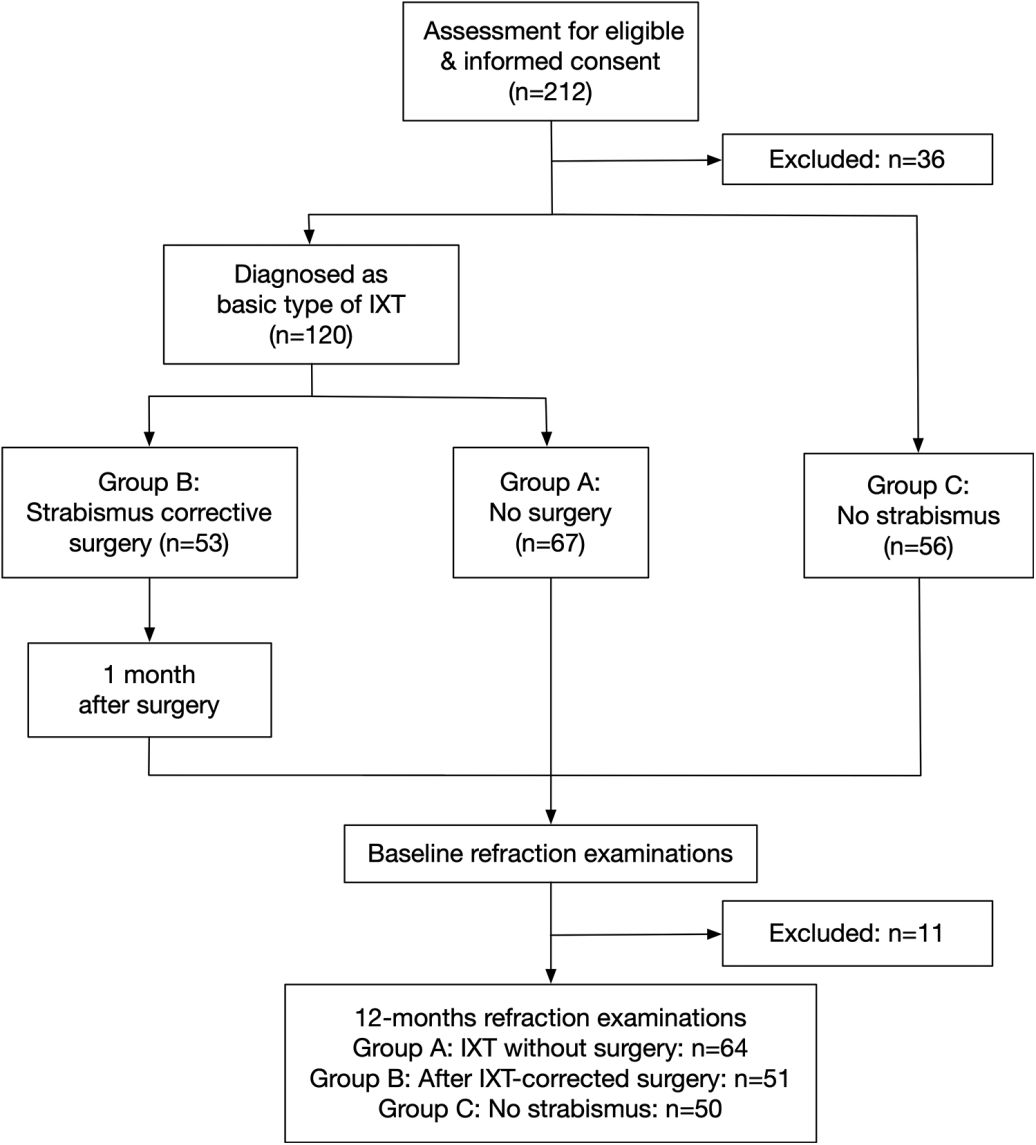
Ophthalmology visits and assessment procedures. IXT, intermittent exotropia.

A total of 176 children aged 4–15 years were recruited for this study. The subjects were divided into three groups: Group A, consisted of 67 patients with basic IXT without surgery; Group B, consisted of 53 patients 1-month after IXT-corrected surgery; and Group C, consisted of 56 patients without any form of strabismus. For group B, patients with basic type of IXT had been successfully treated with unilateral medial rectus plication and lateral rectus recession, and achieved straight eye alignment at 1-month visit after surgery. The baseline clinical examinations were taken at the initial visit for Group A and C, and at 1-month visit after IXT-corrected surgery for Group B (Figure 1). The last refractive examination was needed at 12-month after the baseline examination. The difference between baseline and 12-month was calculated as the parameter at 12-months visit minus the same parameter at baseline (Δ=parameter at 12 months – the same parameter at baseline).

All subjects underwent clinical examinations including exodeviation angle, Newcastle score (NCS) (29) (Supplementary Table S1) and visual function. Professional advice relating to strabismus surgery, spectacles prescription and eye usage habits was given to patients and parents. Parents were instructed to provide fully corrected spectacles for their children, who were required to wear these glasses throughout the day. Subjects were required to be followed for 12 months. A total of 11 patients were excluded from the final analysis due to various reasons, including undergoing strabismus surgery during the follow-up, loss of contact, among others. Consequently, 165 subjects were ultimately included in the analysis (Figure 1).

Written informed consent to participate was obtained from the parents or legal guardians of any participant prior to participation. The study followed the tenets of the Declaration of Helsinki and was approved by the Ethics Committee of Beijing TongRen Hospital (approved number: TRECKY2020-142).

### 2.2 Routine ocular examinations

During each visit, all patients underwent complete ophthalmic examinations and assessments, including the following tests:

#### 2.2.1 Baseline demographic data

Information on gender, age, past medical history, and eye usage habits was collected from parents. To rule out anterior and posterior segment diseases, slit-lamp examinations and fundus color photography were performed. Ocular biometry examination included AL, aqueous depth (AD), lens thickness (LT), flat keratometry (Kf) and steep keratometry (Ks) (Lenstar LS-900; Haag-Streit, Bern, Switzerland).

#### 2.2.2 Refraction examination

For children aged 4–6 years, cycloplegia was induced by 1% atropine ointment (Shenyang Xingqi Eye Medicine Co, Ltd.) twice a day for 3 days; For children aged above 6 years, after corneal anesthesia with 1 drop of topical anesthetic agent (Alcaine, Alcon, Fort Worth, TX), cycloplegia was induced by 2 drops of 1% cyclopentolate (Cyclogyl, Alcon Health care S.A.) and 1 drop of 0.5% tropicamide phenylephrine (Mydrin P, Santen, Osaka, Japan) with an interval of 5 min between drops. The pupillary light reflex and the pupil size were tested thirty minutes after medication application, then cycloplegic refraction (Topcon RM-800, Topcon Corp, Tokyo, Japan) was measured. The refraction was defined as the spherical equivalent refraction (SE; SE = spherical power + cylinder power/2). The myopic shift was defined as the SE changing during 12 months (myopic shift = SE_12months_ − SE_baseline_).

#### 2.2.3 Strabismus examination

The exodeviation angle and NCS (29) (Supplementary Table S1) were evaluated by the same experienced pediatric ophthalmologist (Dr. JF). The alternate prism cover test and cover-uncover test at distance (6 m) and near (33 cm) were performed to assess the exodeviation angle. Patients were asked to accept examinations under full correction of refractive errors after 1 h of monocular occlusion.

#### 2.2.4 Accommodation and convergence function evaluation

The monocular amplitude of accommodation (AMP) was measured with the minus lens technique at a distance of 40 cm (30, 31). The accommodative facility (AMF) was tested by the ±2.0D flip method (32). The accommodation response was measured via the monocular estimation method (MEM) (32). The near point of convergence (NPC) was tested with push-up method (33). The stimulus accommodative convergence to accommodation ratio (AC/A ratio) was tested via the method with synoptophore (34).

### 2.3 Data analysis

Only data from participants who completed the 12-month visit were used for the statistical analysis and only data from right eye were included in the statistical analysis. The statistical analysis was performed using SPSS version 26.0 (SPSS, Inc., Chicago, IL, USA). The normality of the measurements was determined by the Shapiro-Wilk test. Descriptive statistics for normally distributed continuous data are reported as mean ± standard deviation (SD) and non-normally distributed data with median (min–max). A comparison of continuous data among three groups was conducted with the ANOVA test for normally distributed data, and Kruskal-Wallis test for non-normally distributed data. Bonferroni's multiple comparisons tests were used to test differences between each two groups and *P* < .0167 was considered significant. For categorical characteristics, chi-square tests were applied. Parameters were compared between right and left eyes with Pearson correlation analysis. The univariate and multivariate linear analyses were used to evaluate the correlations of baseline SE and AL with various parameters, so were AL elongation and SE progression with parameters changing (Δ). For each analysis, a 2-tailed model was utilized, and *P* < .05 was considered to indicate statistical significance.

## 3 Results

### 3.1 Overall subjects

A total of 165 subjects were included in this study, consisting of 83 male and 82 female, with a mean age of 8.09 ± 2.06 years. In this cohort, 64 patients (38.8%) were identified to have basic type of IXT without IXT-corrected surgeries (Group A), 51 patients (30.9%) were after IXT-corrected surgery (Group B) and 50 subjects (30.3%) did not have any forms of strabismus (Group C). The baseline SE and AL showed no significant differences (SE: *P* = .115; AL: *P* = .303) (Table 2). Likewise, AD, LT, mean keratometry (Km, which was calculated as the mean value of Kf and Ks), and accommodative response were comparable among different groups (all *P* > .05) (Table 2).

**Table 2:** Clinical characteristics of the study population.

Parameters		Overall subjects (*N* = 165)	Group A IXT without surgery (*N* = 64)	Group B After IXT-corrected surgery (*N* = 51)	Group C No strabismus (*N* = 50)	*P*-values
Age at baseline (years)		8.09 ± 2.06	8.28 ± 1.85	7.63 ± 2.25	8.33 ± 2.08	.156**
Sex (Male:Female)		83:82	27:37	24:27	32:18	.060*
Baseline SE (D)		−1.46 ± 1.37	−1.62 ± 1.09	−1.13 ± 1.82	−1.60 ± 1.09	.115**
Baseline AL (mm)		23.93 ± 1.04	24.02 ± 0.97	23.74 ± 1.23	24.00 ± 0.89	.303**
12-months exodeviation angle (PD)	33 cm	–	−39.45 ± 15.61	−7.65 ± 9.59	–	**<.001** ***
	6 m	–	−34.92 ± 15.34	−6.80 ± 8.78	–	**<.001** ***
12-months NCS		–	–	0.84 ± 1.50	–	**<.001** ***
AD (mm)		3.21 ± 0.25	3.17 ± 0.26	3.24 ± 0.23	3.23 ± 0.24	.251**
LT (mm)		3.39 ± 0.16	3.40 ± 0.16	3.38 ± 0.15	3.39 ± 0.16	.773**
Km (D)		43.45 ± 1.49	43.29 ± 3.62	43.59 ± 1.34	43.54 ± 1.46	.502**
AMP (D)		8.38 ± 2.43	8.84 ± 1.96	8.59 ± 2.66	7.58 ± 2.57	**.017** **
Group A vs. Group B		–	–	–	–	.585****
Group B vs. Group C		–	–	–	–	.034****
Group A vs. Group C		–	–	–	–	**.006** ****
AMF (cpm)		7.35 ± 2.71	8.11 ± 2.51	6.53 ± 2.72	7.21 ± 2.74	**.007** **
Group A vs. Group B		–	–	–	–	**.002** ****
Group B vs. Group C		–	–	–	–	.197****
Group A vs. Group C		–	–	–	–	.073****
Accommodative response (D)		0.73 ± 0.24	0.72 ± 0.19	0.71 ± 0.32	0.78 ± 0.18	.233**
NPC (mm)		8.35 ± 4.06	8.20 ± 3.76	9.59 ± 4.88	7.36 ± 3.64	**.021** **
Group A vs. Group B		–	–	–	–	.070****
Group B vs. Group C		–	–	–	–	**.006** ****
Group A vs. Group C		–	–	–	–	.265****
AC/A ratio (PD/D)		2.46 ± 1.55	2.97 ± 1.77	2.21 ± 1.51	2.05 ± 1.06	**.003** **
Group A vs. Group B		–	–	–	–	**.010** ****
Group B vs. Group C		–	–	–	–	.585****
Group A vs. Group C		–	–	–	–	**.001** ****
Time spending on near work (hours/day)		5.56 ± 2.75	5.91 ± 2.37	4.99 ± 3.12	5.86 ± 2.62	.181**
Time spending on outdoor activities (hours/day)		1.86 ± 1.26	1.58 ± 0.82	2.15 ± 1.69	1.83 ± 1.00	.079**

Significant factors appear in boldface. Continuous variables are reported as mean ± SD.

For Group B, baseline clinical examinations were taken at 1-month visit after strabismus-corrected surgery. For Group A and C, same examinations were taken at the initial visit.

IXT, intermittent exotropia; SE, spherical equivalent; D, diopters; AL, axial length; PD, prism diopters; NCS, Newcastle score; AD, aqueous depth; LT, lens thickness; Km, mean keratometry; AMP, accommodative amplitude; AMF, accommodative facility; cpm, cycles per minute; NPC, near point of convergence; AC/A ratio, accommodative convergence to accommodation ratio.

* χ^2^ test.

** ANOVA.

*** Independent *t*-test.

**** Parametric *post hoc* test (Bonferroni's test).

For all participants diagnosed as basic type of IXT at initial visit (Group A and B, *n* = 120), Group A and Group B (before surgery) shared comparable exodeviation angles (−38.57 ± 12.68PD vs. −43.80 ± 17.31PD, *P* = .067; −36.35 ± 13.42PD vs. −41.80 ± 17.35PD, *P* = .062) and NCS (4.06 ± 1.58 vs. 4.61 ± 1.88, *P* = .094). Patients in Group B achieved straight eye alignment at 1-month visit after surgery (referred as baseline). At 12-months visit, the exodeviation angles (−7.65 ± 9.59PD vs. −39.45 ± 15.61PD; −6.80 ± 8.78PD vs. −34.92 ± 15.34) and NCS (0.84 ± 1.50 vs. 3.92 ± 1.51) in Group B both showed significant less than those in Group A (all *P* < .001) (Table 2).

### 3.2 Differences in myopic shift and AL elongation among groups

**Figure 2:**
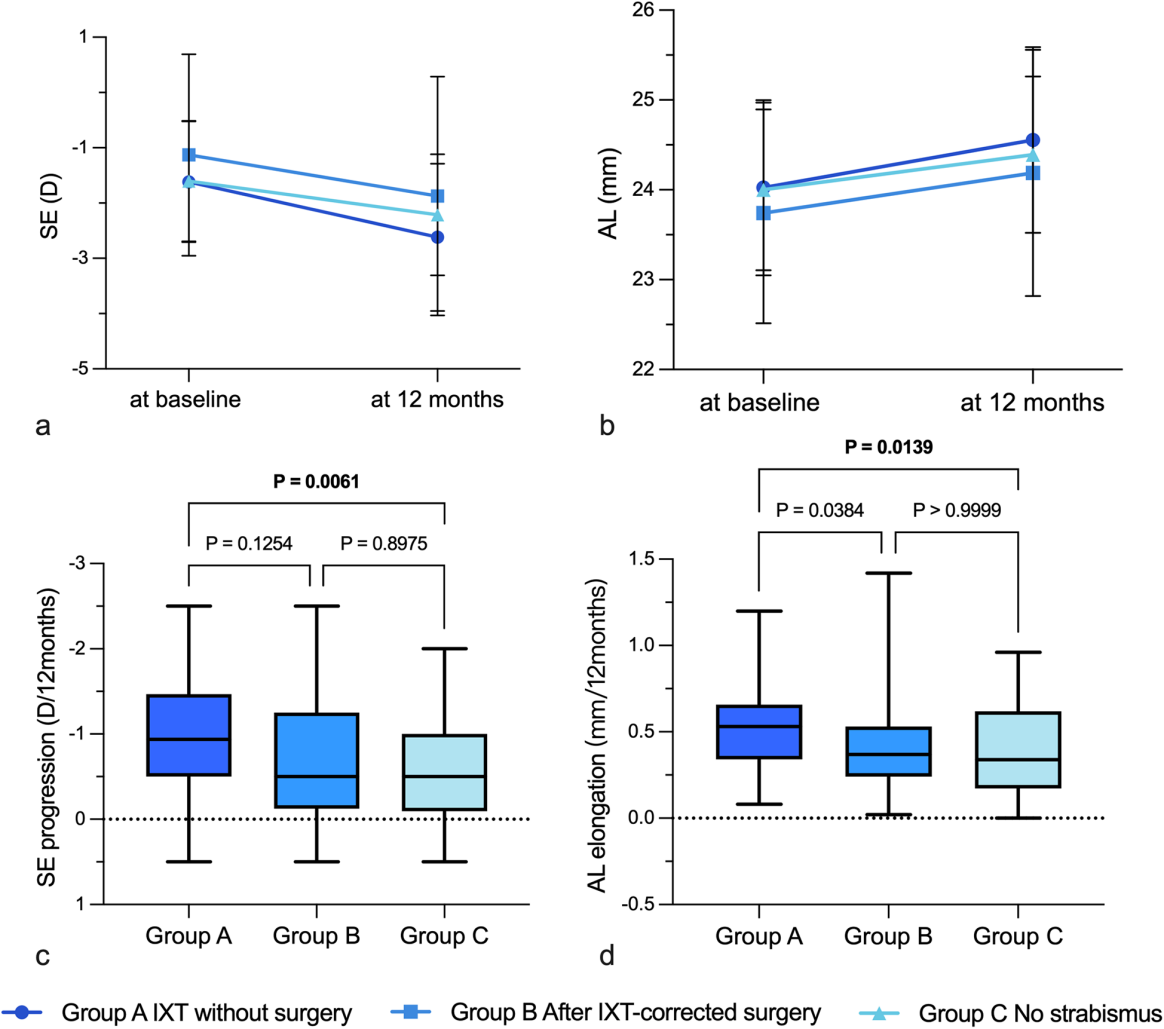
Se progression and AL elongation in different groups during 12 months. **(a)** SE progression in different groups; **(b)** AL elongation in different groups; **(c)** Differences in SE progression among different groups; **(d)** Differences in AL elongation among different groups. IXT, intermittent exotropia; D, diopters; AL, axial length; SE, spherical equivalent.

Table 3 and Figure 2 showed the statistical differences in SE changes (*P* = .007) and AL elongation (*P* = .007) among three groups. Group A tended to have greater SE progression (−1.00 ± 0.61D) and faster AL elongation (0.53 ± 0.24 mm) during 12 months than Group C (−0.60 ± 0.63D and 0.39 ± 0.25 mm) (*P* = .006 and *P* = .014, respectively). Although SE progression and AL elongation for Group B [−0.74 ± 0.80D and 0.37 (0.02–1.42)mm] was less than that in Group A (−1.00 ± 0.61D and 0.53 ± 0.24 mm), this was not found to be statistically significant (*P* = .125 and *P* = .038, respectively) (Table 3, Figure 2).

**Table 3:** Se progression and AL elongation in different groups during 12 months.

Parameters	Group A IXT without surgery (*N* = 64)	Group B After IXT-corrected surgery (*N* = 51)	Group C No strabismus (*N* = 50)	*P*-values			
				Among 3 groups	Between Group A and B	Between group B and C	Between group A and C
SE progression (D/12 months)	−1.00 ± 0.61	−0.74 ± 0.80	−0.60 ± 0.63	**.0065** *	.1254***	.8975***	**.0061** ***
AL elongation (mm/12 months)	0.53 ± 0.24	0.37 (0.02–1.42)	0.39 ± 0.25	**.0070** **	.0384***	>.9999***	**.0139** ***

Significant factors appear in boldface.

Continuous variables are reported as mean ± SD or median (min–max).

SE, spherical equivalent; D, diopters; AL, axial length; IXT, intermittent exotropia.

* ANOVA.

** Kruskal-Wallis test.

*** Parametric *post hoc* test (Bonferroni's test).

### 3.3 Differences in accommodation- and convergence-related parameters among groups

Statistical differences were observed in AMP (*P* = .017), AMF (*P* = .007), NPC (*P* = .021), and AC/A ratio (*P* = .003) among three groups, as detailed in Table 2. Group A exhibited significantly higher AMPs (8.84 ± 1.96D) and AC/A ratios (2.97 ± 1.77PD/D) compared to Group C (7.58 ± 2.57D and 2.05 ± 1.06PD/D) (*P* = .006 and *P* = .001, respectively). Additionally, their AMFs (8.11 ± 2.51 cpm) and AC/A ratio (2.97 ± 1.77PD/D) were significantly greater than those in Group B (6.53 ± 2.72 cpm, 2.21 ± 1.51PD/D; *P* = .002, *P* = .010).

### 3.4 Associations between baseline refraction and accommodation- and convergence-related parameters

For IXT children without strabismus-corrected surgery (Group A), the baseline SE was significantly correlated with gender (*R* = 0.330, *P* = .008), LT (*R* = 0.267, *P* = .041) according to univariate linear analysis (Table 4). In the multivariate model, significant negative relationship was observed between AMF and baseline SE after adjusting for age and gender (*β* = −0.091, *P* = .040). The baseline AL showed associations with gender (*R* = 0.398, *P* = .001), AD (*R* = 0.421, *P* = .001) and Km (*R* = 0.719, *P* < .001) according to univariate linear analysis while the associations with AD (*β* = 1.192, *P* = .007), Km (*β* = −0.409, *P* < .001) and accommodative response (*β* = 0.796, *P* = .040) were significant according to multivariate linear analysis.

**Table 4:** Associations between baseline SE and AL and baseline testing parameters for IXT children without surgery.

Baseline parameters	Univariate analysis			Multivariate analysis*		
	R	Beta (95% CI)	*P*-value	Beta (95% CI)	Standardized beta	*P*-value
For baseline SE (D) (*N* = 64)						
Gender	0.325	0.714 (0.187, 1.240)	**.009**	–	–	–
Age (years)	0.212	−0.125 (−0.272, 0.021)	.092	–	–	–
Average exodeviation angle (PD)	0.058	−0.005 (−0.027, 0.017)	.654			
NCS	0.070	0.048 (−0.131, 0.227)	.591			
AD (mm)	0.250	−1.030 (−2.095, 0.035)	.058	−0.647 (−1.676, 0.381)	−0.156	.213
LT (mm)	0.267	1.804 (0.074, 3.535)	**.041**	1.0171 (−0.694, 2.836)	0.158	.229
Km (D)	0.083	0.056 (−0.115, 0.226)	.516			
AMP (D)	0.020	−0.005 (−0.071, 0.061)	.878			
AMF (cpm)	0.208	−0.079 (−0.173, 016)	.101	−0.091 (−0.178, −0.004)	−0.242	**.040**
Accommodative response (D)	0.114	−0.438 (−1.417, 0.542)	.375			
NPC (cm)	0.056	−0.014 (−0.077, 0.050)	.666			
AC/A ratio (PD/D)	0.166	0.077 (−0.043, 0.197)	.205			
For baseline AL (mm) (*N* = 64)						
Gender	0.398	−0.780 (−1.236, −0.324)	**.001**	–	–	–
Age (years)	0.188	0.099 (−0.032, 0.230)	.136	–	–	–
Average exodeviation angle (PD)	0.037	0.003 (−0.017, 0.022)	.773			
NCS	0.103	0.065 (−0.096, 0.226)	.424			
AD (mm)	0.421	1.564 (0.700, 2.427)	**.001**	1.192 (0.338, 2.045)	0.321	**.007**
LT (mm)	0.238	−1.427 (−2.975, 0.120)	.070	−0.659 (−2.196, 0.878)	−0.110	.394
Km (D)	0.719	−0.432 (−0.538, −0.326)	**<.001**	−0.409 (−0.501, −0.318)	−0.681	**<.001**
AMP (D)	0.012	0.004 (−0.087, 0.095)	.928			
AMF (cpm)	0.035	0.011 (−0.072, 0.095)	.787			
Accommodative response (D)	0.195	0.653 (−0.189, 1.494)	.126	0.796 (0.039, 1.553)	0.238	**.040**
NPC (cm)	0.006	0.001 (−0.053, 0.056)	.961			
AC/A ratio (PD/D)	0.013	−0.006 (−0.120, 0.108)	.919			

Significant factors appear in boldface. Continuous variables are reported as mean ± SD.

Average exodeviation angle, mean value of exotropia prism diopters at 33 cm and 6 m.

IXT, intermittent exotropia; SE, spherical equivalent; D, diopters; PD, prism diopters; NCS, Newcastle score; AL, axial length; AD, aqueous depth; LT, lens thickness; Km, mean keratometry; AMP, accommodative amplitude; AMF, accommodative facility; cpm, cycles per minute; NPC, near point of convergence; AC/A ratio, accommodative convergence to accommodation ratio.

* Adjusted for gender and age.

### 3.5 Associations between refractive changes and deviation angle changes

As shown in Table 5, the SE progression during 12 months was significantly correlated with the changes of exodeviation angles according to both univariate (*R* = 0.266, *P* = .035) and multivariate (*β* = 0.010, *P* = .041) linear analysis, so was the AL elongation (*β* = −0.005, *P* = .026). Each one prism diopter (PD) increase in the exodeviation angles was correlated with a 0.01D SE progression and a 0.005 mm AL elongation during the 12-month follow-up.

**Table 5:** Associations between the myopia shift and testing parameters’ changing for IXT children without surgery.

Parameters changing	Univariate analysis			Multivariate analysis*		
	R	Beta (95% CI)	*P*-value	Beta (95% CI)	Standardized beta	*P*-value
For SE progression (D/12 months) (*N* = 64)						
Δ Average exodeviation angle (PD)	0.266	0.011 (0.001, 0.020)	**.035**	0.010 (0.000, 0.020)	0.259	**.041**
Δ NCS	0.215	−0.065 (−0.140, 0.011)	.093	−0.063 (−0.139, 0.013)	−0.210	.102
Δ AD (mm)	0.017	0.065 (−0.900, 1.030)	.894			
Δ LT (mm)	0.078	0.489 (−1.191, 2.169)	.562			
Δ Km (D)	0.047	−0.086 (−0.556, 0.383)	.714			
Δ AMP (D)	0.011	0.002 (−0.035, 0.038)	.928			
Δ AMF (cpm)	0.092	0.012 (−0.022, 0.046)	.475			
Δ Accommodative response (D)	0.010	−0.013 (−0.371, 0.344)	.941			
Δ NPC (cm)	0.063	−0.006 (−0.031, 0.019)	.629			
Δ AC/A ratio (PD/D)	0.198	0.035 (−0.011, 0.081)	.136	0.043 (−0.003, 0.088)	0.242	.067
For AL elongation (mm/12 months) (*N* = 64)						
Δ Average exodeviation angle (PD)	0.294	−0.006 (−0.011, −0.001)	**.020**	−0.005 (−0.010, −0.001)	−0.270	**.026**
Δ NCS	0.088	0.013 (−0.025, 0.051)	.498			
Δ AD (mm)	0.055	0.103 (−0.380, 0.587)	.671			
Δ LT (mm)	0.157	−0.512 (−1.383, 0.360)	.244			
Δ Km (D)	0.081	0.075 (−0.160, 0.310)	.526			
Δ AMP (D)	0.106	0.008 (−0.011, 0.026)	.404			
Δ AMF (cpm)	0.030	−0.002 (−0.019, 0.015)	.817			
Δ Accommodative response (D)	0.013	−0.009 (−0.189, 0.171)	.919			
Δ NPC (cm)	0.209	0.010 (−0.002, 0.022)	.106	0.009 (−0.003, 0.021)	0.189	.138
Δ AC/A ratio (PD/D)	0.051	−0.005 (−0.029, 0.020)	.704			

Significant factors appear in boldface. Continuous variables are reported as mean ± SD.

Average exodeviation angle, mean value of exotropia prism diopters at 33 cm and 6 m.

Δ equals to parameter at 12-months visit minus the same parameter at baseline.

IXT, intermittent exotropia; SE, spherical equivalent; PD, prism diopters; NCS, Newcastle score; AL, axial length; AD, aqueous depth; LT, lens thickness; Km, mean keratometry; D, diopters; AMP, accommodative amplitude; AMF, accommodative facility; cpm, cycles per minute; NPC, near point of convergence; AC/A ratio, accommodative convergence to accommodation ratio.

* Adjusted for gender and age.

## 4 Discussion

In this prospective study, we compared the SE progression and AL elongation over a 12-month follow-up period in children with basic type of IXT, post-IXT-corrected surgery and without strabismus. Our findings indicated that children with basic type of IXT experienced significantly higher annual rates of SE progression and AL elongation compared to normal controls. Although children who had received IXT-corrected surgery demonstrated a slower rate of myopic shift compared to children without strabismus-corrected surgery, the difference was no statistically significant. The present study also incorporated accommodation- and convergence-related parameters to identify potential clinical indicators of myopic shift among children with strabismus. Notably, the degree of myopic shift was positively correlated with the exodeviation angle development in children with basic type of IXT. This correlation could be pivotal in identifying children at higher risk of rapid myopic shift in the future. This study is the first to explore the relationship between myopic shift and strabismus development in children with IXT, emphasizing the role of accommodation and convergence.

For nonstrabismus children (Group C), the mean annual myopic shift was −0.60 ± 0.63D and axial elongation was 0.39 ± 0.25 mm, which were greater than data previously reported in several clinical trials. In a randomized clinical trial for Indian children aged 6–14 years, the mean annual progression for SE was −0.35 ± 0.4D and AL 0.28 ± 0.28 mm (35). In Spain in 2016, one clinical trial reported the mean refractive progression and AL elongation of children aged 8–12 years was −0.55 ± 0.45D and 0.21 ± 0.10 mm per year (36). The current SE progression and AL elongation towards myopia was faster, partly because of the enrollment of premyopic children in this study. Mutti et al. (37) and Xiang et al. (38) reported that the refraction and AL change faster in premyopic children and gradually slows down after myopia development. The current group C included 6 children with premyopia and 44 with myopia. The rate of SE progression was slightly higher in premyopoes (−0.79 ± 0.89D) than myopes (−0.58 ± 0.59D) while they shared comparable AL elongation (0.39 ± 0.30 mm vs. 0.39 ± 0.25 mm) during 12-month follow-up. In addition, relatively younger age might be one of explanation for faster myopic shift than what were reported before (39). Recently, several clinical trials (40, 41) targeting Asian children showed comparable or even faster myopic shift with this study, which may be reflective of a trend of faster myopic shift in Asian countries in recent years.

The rate of myopic shift in Group A was significantly greater than that in Group C (SE progression: *P* = .006; AL elongation: *P* = .014). The finding was in consistent with the result of a population-based study conducted by Ekdawi et al. (15). They reported that the Kaplan-Meier rate of developing myopia in IXT children was 7.4% by 5 years of age, 46.5% by 10 years, and 91.1% by 20 years in the United States. This was significantly higher compared to similarly aged American children (42–44). Recent researches have focused more on refractive progression in IXT children, and those studies aimed to investigate the myopic shift in IXT patients and compare it with that of myopia alone (16, 17, 20). Shin et al. (16) and Kim et al. (17) suggested that the progression was comparable, while Oruz et al. (20) believed that the progression was significantly greater in children with IXT than in the normal population. Both IXT and normal groups in researches of Shin (16) and Kim (17) were consisted of children who have already myopic (≤−0.50D). Confounding factors which may influence myopic shift including family history, near work or outdoor activity were not considered and controlled in all three researches (16, 17, 20). This study tried to remedy the limitations and found some differences. The current results have suggested that SE progression in patients with IXT may not be the same as those in patients with myopia alone. Premyopic children were enrolled to study the refraction of broader population. To eliminate confounding factors, the study excluded children with a family history of high myopia and limited myopia interventions. Subjects' time spent on near work (*P* = .181) and outdoor activities (*P* = .079) also showed no significant differences among groups (Table 2). However, parents of children with IXT tend to impose stricter time limitations on near work than parents of children with myopia alone in clinical practice, which is because strabismus is considered as a comparatively rare and serious condition. This may have obscured a possible relationship between IXT and myopic shift in previous studies. In addition, we analyzed AL elongation as a supplementary and objective indicator of refractive progression. AL could be treated as a parameter or an endophenotype of myopia, particularly for developing premyopia (5).

The greater axial elongation observed in IXT children (Group A) might be associated with the over-accommodation. It is not clear if the increased convergence necessary to control the exodeviation brings along over-accommodation (45) or if the opposite happens, that is, the control of the deviation is mainly warranted by accommodation through the AC/A ratio (23, 24). But it is generally believed that in IXT children, an increase in accommodation contributes to maintain ocular alignment. As the parameter represents maximum ability of the eye to change its refractive power from the relaxed state by accommodating when fixating on a near target (46), AMPs in IXT children showed significantly greater than normal controls, which partially reflected the accommodative ability in the current study. Greater AMPs have also been reported as the clinical indicators for faster myopia progression in IXT children (47). Several studies (25–27) have shown that the eye undergoes a transient period of axial elongation on the axis after brief periods of sustained accommodation. One hypothesis (48, 49) suggests that the accommodative ciliary muscle applies an internal mechanical force upon the globe, which decreases the scleral and choroidal equatorial circumference. As a result, axial elongation is the only way to maintain the globe volume. Persistent retinal defocus induced by the circumstances then causes the shift to permanent myopia (50, 51). In addition, high order aberrations (HOAs) consistently increase with greater accommodative demands (52, 53), which was caused by near work or attempt to align both eyes in IXT. The increase in HOA resulted in poor retinal image quality at distance and near (54), which provides a potential mechanism for the reported link between myopic shift and binocular visual functions.

However, the hypothesis about increased accommodation demand contributes to observed greater myopic shift is not without controversies. There is no consensus on if excess accommodative demand leads to faster permanent axial elongation, or if the opposite happens, that is, reduced accommodative demand contributes to slower myopic shift in IXT children. The debate on whether the overminus lens therapy, which was an effective non-surgical treatment for IXT, accelerated myopic shift related to accommodation stimulation was recently been discussed (55–57). It is also unclear if the strabismus-corrected surgery, which theoretically reduces the accommodative effort required for alignment could moderate myopic shift (15–17). In the present study, it was found that the only parameter related to both SE progression and axial elongation was the changing of exodeviation angle, rather than the changing of one single accommodative or convergent parameter. It has been suggested that myopia may lead to a lower accommodative demand resulting in reduced convergence and therefore the development of IXT. Alternatively, the development of IXT may be caused by lower convergence ability, resulting in higher accommodative demand but lower accommodative ability, and therefore the myopic shift. The relationship between accommodation or convergence and myopic shift may have been obscured due to the lack of a single testing parameter that can accurately represent the actual accommodation demand or ability or the gap between them.

The study found that the annual rate of myopic shift in Group B was slower than Group A [−0.74 ± 0.80D vs. −1.00 ± 0.61D; 0.37 (0.02–1.42)mm vs. 0.53 ± 0.24 mm]. However, there was no significant differences in both SE progression (*P* = .125) and AL elongation (*P* = .038) between two groups. These findings are in consistent with prior analysis (15–17). However, the treatment of IXT-surgery in previous studies was not randomized, in which worse-controlled IXT children were divided into surgery group and well-controlled ones into observed group. The comparable exodeviation angles and NCS between Group A and B could control the bias to some extent in the present study. Theoretically, surgery reduces the accommodative effort required for ocular alignment for IXT children. But the result about the corresponding reduced myopic shift was not provided. It is still unclear whether the surgical removal of IXT is relax or disturbance for the accustomed accommodative condition. Nevertheless, surveys from parents showed that children who underwent IXT corrected surgeries spent the least amount of time on near work per day (4.99 ± 3.12 h) and the most amount of time on outside activities (2.15 ± 1.69 h). However, there were no significant differences among groups (*P* = .181; *P* = .079). This phenomenon could be explained by the increased attention parents paid to their children after the surgeries in order to prevent strabismus relapse. However, we believe that the limited sample size and study period may have obscured a possible relationship, as the magnitude of the difference in annual myopic shift between surgeries accepted (−0.74D) and nonstrabismus (−0.60D) was much smaller than that between IXT with (−0.74D) and without surgeries (−1.00D). Moreover, the difference of binocular accommodation and convergence before and after strabismus-corrected surgery has been seldom studied. Therefore, future research with a larger sample size and longer-term follow-up is needed to determine the impact of surgeries on IXT more clearly. If necessary, make comparisons between the myopic shift, accommodation- and convergence-related binocular functions in children with IXT before and after surgeries.

This study has some limitations. Firstly, the follow-up period was only 12months, which may not be sufficient for observing the long-term myopic shift. However, previous studies have shown that young myopic children experience drastic elongation of annual axial length (58), and myopia tends to stabilize by age of 18 (59, 60). Additionally, the children in all groups were of the same age (*P* = .156) in this study. Thus, the variation tendency of refractions remained valid even with a shorter follow-up duration. Moreover, while each parent provided the information about child's eye usage and major influencing factors were analyzed, not all factors relevant to myopic shift were stringently controlled, which can be challenging in a clinical setting. Potential confounding variables related to myopic progression such as high myopia, high myopic family history or accepting myopic control and treatment were excluded to clarify the association with IXT and associative functions. The population should be included in future researches as the percentage is increasing not only in IXT children but also in children without any forms of strabismus. Finally, the sample size was relatively limited, which possibly conceal the significant differences, especially the difference in myopic shift between IXT children without surgery and after surgery. Future research with larger sample sizes, broader enrollment of population and longer-term follow-up should be conducted to more clearly determine the relationship between myopia and intermittent exotropia.

## 5 Conclusions

Children with IXT tend to show more myopic shift than patients without strabismus. Surgical correction of strabismus seems to moderate the myopic shift, although this effect is not significant. The rate of myopic shift was positively correlated with the development of exodeviation angle in IXT children, highlighting the importance of addressing binocular vision abnormalities in myopia management.

## Data Availability

The data that support the findings of the current study are not publicly available due to containing information that could compromise the privacy of research participants, but are available from the corresponding author Jing Fu.
