# Supplementary material for: Effects of basic type of intermittent exotropia on myopic shift in children: a 12-month observational study
Source: Front Pediatr. 2025 Jan 6;12:1513062. doi: 10.3389/fped.2024.1513062 (PMC11743707; doi:10.3389/fped.2024.1513062)
Supplement: Supplementary file 1 [file Table1.docx]

Supplementary Table. Components of Newcastle Control Score (NCS) (29)

| **Score** | **Component** |
| --- | --- |
| **Home control** | Exotropia or monocular eye closure seen: |
| 0 | Never |
| 1 | <50% of time fixing in distance |
| 2 | >50% of time fixing in distance |
| 3 | >50% of time fixing in distance + seen at near |
| **Clinic control for near** | |
| 0 | Immediate realignment after dissociation |
| 1 | Realignment with aid of blink or re-fixation |
| 2 | Remains manifest after dissociation/prolonged fixation |
| 3 | Remains manifest after dissociation/prolonged fixation |
| **Clinic control for distance** | |
| 0 | Immediate realignment after dissociation |
| 1 | Realignment with aid of blink or re-fixation |
| 2 | Remains manifest after dissociation/prolonged fixation |
| 3 | Remains manifest after dissociation/prolonged fixation |
| **Total NCS: (Home + Near + Distance) =** | |

Reference:

29. Buck D, Clarke MP, Haggerty H, Hrisos S, Powell C, Sloper J, et al. Grading the severity of intermittent distance exotropia: the revised Newcastle Control Score. Br J Ophthalmol. 2008;92(4):577.
